# Supplementary material for: Chemical and Quality Analysis of Beauty Tea Processed from Fresh Leaves of Tieguanyin Variety with Different Puncturing Degrees
Source: Foods. 2023 Apr 22;12(9):1737. doi: 10.3390/foods12091737 (PMC10178084; doi:10.3390/foods12091737)
Supplement: Supplementary file 1 [file foods-12-01737-s001.zip › Table S1.pdf]

Table S1. The information on 95 non-volatile differential metabolites in fresh tea leaves.

metabolite name	TR (min)	accurate mass	theoretical mass	ppm	MS/MS ions	formula	Ref.
**Catechins and their derivatives**							
8-C-Ascorbylepigallocatechin 3-gallate	4.57	631.0938	631.0941	-0.43	163.0037,495.0568	$C_{28}H_{24}O_{17}$	
(-)-Epigallocatechin	4.60	306.0735	305.0667	-1.36	305.0664	$C_{15}H_{14}O_{7}$	
(−)-Gallocatechin	5.76	306.0739	305.0667	-0.25	125.0243,305.0665	$C_{15}H_{14}O_{7}$	
Epigallocatechin gallate	5.77	457.0776	457.0776	0.01	169.0141,125.0243,305.0664	$C_{22}H_{18}O_{11}$	
Epicatechin 3-glucoside	8.26	452.1319	451.1246	0.04	271.0607,169.0142,125.0244,289.0717	$C_{21}H_{24}O_{11}$	
3,5-Digalloylepicatechin	8.26	593.0930	593.0937	-1.12	423.0721,441.0824,593.0940	$C_{29}H_{22}O_{14}$	
Epicatechin-(4beta->8)-gallocatechin	4.41	593.1289	593.1301	-1.98	305.0663,441.0827	$C_{30}H_{26}O_{13}$	
Gallocatechin-(4alpha->8)-epicatechin	4.65	593.1288	593.1301	-2.17	407.0770,289.0714,441.0823	$C_{30}H_{26}O_{13}$	
Epigallocatechin-(4beta->8)-epicatechin 3-O-gallate	5.64	745.1394	745.1410	-2.23	407.0769,467.0980,593.1289	$C_{37}H_{30}O_{17}$	
ent-Epicatechin-(4alpha->8)-ent-epicatechin 3'-gallate	5.66	775.1492	729.1461	-3.21	407.0768,577.0983,289.0714	$C_{37}H_{30}O_{16}$	
Theasinensin A	5.81	913.1451	913.1469	-2.02	457.0774,169.0142,125.0243,305.0666	$C_{44}H_{34}O_{22}$	
Epigallocatechin 3-O-gallate-(4beta->6)-epicatechin 3-O-gallate	6.17	898.1615	897.1520	2.47	745.1389,897.1498,727.1283,559.0880	$C_{44}H_{34}O_{21}$	
**Flavones and flavonols/their glycoside**							
5-hydroxypseudobaptigenin	2.46	343.0461	297.0405	0.68	169.0141,170.0221,177.0194	$C_{16}H_{10}O_{6}$	
(2S)-dihydrotricetin	3.49	304.0585	303.0510	0.57	125.0242,305.0663,137.0242,165.0190,167.0346	$C_{15}H_{12}O_{7}$	
Quercetin 3-(3-p-coumaroy	3.78	609.1235	609.1250	-2.40	423.0718,305.0665,407.0774,125.0244	$C_{30}H_{26}O_{14}$	

lglucoside)						
Myricetin 7-(6''-galloylglucoside)	4.77	631.0941	631.0941	-0.00	271.0454,169.0141,313.0563,211.0242	$C_{28}H_{24}O_{17}$
Eriodictyol 7-(6-galloylglucoside)	4.98	601.1201	601.1199	0.41	124.0164,347.0778,183.0296,449.0981	$C_{28}H_{26}O_{15}$
Isorhamnetin 4'-O-glucuronide	5.36	473.0724	491.0831	-0.29	303.0511,285.0402,321.0614,473.0742	$C_{22}H_{20}O_{13}$
Eriocitrin	5.86	595.1658	595.1668	-1.80	401.0761,327.0515,473.1082,433.1350	$C_{27}H_{32}O_{15}$
Safflor Yellow A	5.87	593.1501	593.1512	-1.81	593.1503,357.0862,453.1027,191.0559	$C_{27}H_{30}O_{15}$
6''-O-Galloylquercimeritrin	5.91	615.1002	615.0992	1.67	559.1239,327.0514	$C_{28}H_{24}O_{16}$
Quercetin 7-glucuronide 3-rhamnoside	6.09	605.1137	623.1254	-1.74	453.1029,591.1140,573.1028	$C_{27}H_{28}O_{17}$
Prunin 6''-O-gallate	6.14	585.1250	585.1250	-0.02	465.0670,169.0141,313.0565	$C_{28}H_{26}O_{14}$
Quercetin 3-[rhamnosyl-(1->2)-alpha-L-arabinopyranoside]	6.35	625.1400		-1.78	625.1399	$C_{26}H_{28}O_{15}$
Myricetin 3-galactoside	6.36	480.0901	479.0831	-0.66	316.0222	$C_{21}H_{20}O_{13}$
Kaempferol 3-rhamnoside 7-galacturonide	6.75	589.1193	607.1305	-1.05	413.0873	$C_{27}H_{28}O_{16}$
Hydroxysafflor yellow A	6.75	593.1503	611.1618	-1.45	593.1497,413.0873,293.0453	$C_{27}H_{32}O_{16}$
Scoparin 2''-xyloside	6.83	594.1574	593.1512	-1.77	413.0875,593.1497,293.0453	$C_{27}H_{30}O_{15}$
Hibiscitrin	6.89	477.0673	495.0780	-0.42	315.0146,169.0141,125.0243,477.0673	$C_{21}H_{20}O_{14}$
Kaempferol 3-arabinofuranoside 7-rhamnofuranoside	6.92	563.1401	563.1406	-0.87	169.0141,563.1403	$C_{26}H_{28}O_{14}$
Isoengelitin	6.94	415.1027	433.1140	-1.74	125.0242,269.0457,287.0562,293.0457	$C_{21}H_{22}O_{10}$
Galangin 3-[galactosyl-(1->	7.04	578.1628	577.1563	-1.34	609.1450,577.1552,413.0875,293.0453	$C_{27}H_{30}O_{14}$

4)-rhamnoside]						
Kaempferol 3-gentiobioside 7-rhamnoside	7.05	756.2093	755.2040	-2.58	755.2026,609.1449,577.1551,413.0874	$C_{33}H_{40}O_{20}$
Plantagoside	7.09	465.1033	465.1039	-1.20	313.0918,151.0037,465.1017	$C_{21}H_{22}O_{12}$
2''-O-Acetylisoorientin	7.31	471.0931	489.1039	-0.42	305.0668,161.0245,327.0518,401.0887,413.0888	$C_{23}H_{22}O_{12}$
Astilbin	7.35	449.1088	449.1089	-0.36	285.0401,287.0552,286.0436,151.0038	$C_{21}H_{22}O_{11}$
Limocitrin 3-glucoside	7.35	489.1039	507.1144	0.16	285.0401,287.0552,286.0436,151.0038	$C_{23}H_{24}O_{13}$
Isorhamnetin 3-(2G-apiosylrutinoside)	7.35	756.2098	899.1583	-2.03	755.2021,285.0401	$C_{33}H_{40}O_{20}$
Kaempferol 3-O-alpha-L-rhamnofuranoside	7.52	477.1038	431.0984	-0.08	169.0143	$C_{21}H_{20}O_{10}$
6''-(4-Carboxy-3-hydroxy-3-methylbutanoyl)hyperin	7.64	589.1192	607.1305	-1.15	300.0275,437.1089	$C_{27}H_{28}O_{16}$
NICTOFLORIN	7.68	593.1498	593.1512	-3.86	593.1503,285.0399,284.0323,447.0929	$C_{27}H_{30}O_{15}$
Carthamone	7.69	448.1003	447.0933	-0.58	285.0397,593.1501,284.0322,447.0933	$C_{21}H_{20}O_{11}$
Astragalin	7.93	447.0931	447.0933	-0.41	284.0324,255.0294,227.0347	$C_{21}H_{20}O_{11}$
Naringin	8.20	579.1719	579.1719	-0.12	579.1714,459.1144	$C_{27}H_{32}O_{14}$
Isolariciresinol 4'-O-beta-D-glucoside	8.35	522.2099	521.2028	-0.33	341.1393,279.0876	$C_{26}H_{34}O_{11}$
Kaempferol 3-(6-acetylgalactoside)	8.43	489.1031	489.1039	-1.43	284.0324,489.1038,255.0299	$C_{23}H_{22}O_{12}$
2'-C-Methylmyricetin 3-rhamnoside 5'-gallate	8.43	629.1143	629.1148	-0.79	284.0322,285.0395,169.0147,489.1036,629.1141	$C_{29}H_{26}O_{16}$
Kaempferol 3-neohesperidoside-7-(2''-p-coumaryllaminaribioside)	9.37	531.1442	1063.2936	1.90	755.1829,901.2385,431.0979,145.0293	$C_{48}H_{56}O_{27}$
Quercetin 3-(6-[4-glucosyl-	9.65	917.2340	917.2357	-1.85	285.0402,885.2441,739.1860,431.0981,145.0294	$C_{42}H_{46}O_{23}$

p-coumaryl]glucosyl)(1->2)-rhamnoside							
Kaempferol 2G-coumaroylrutinoside	9.80	739.1859	739.1880	-2.85	285.0403,593.1306,739.1868	$C_{36}H_{36}O_{17}$	
Kaempferol 3-neohesperidoside-7-(2''-p-coumarylglucoside)	10.42	901.2383	901.2408	-2.74	901.2395,635.1418	$C_{42}H_{46}O_{22}$	
Hordatine B	15.43	561.2918	579.3049	-4.41	487.2386,488.2390	$C_{29}H_{40}N_8O_5$	
**Phenolic acids**							
Glucinol	0.75	342.1163	341.1089	0.15	179.0558,311.0982,161.0454	$C_{12}H_{22}O_{11}$	
Chlorogenic acid	4.21	353.0880	353.0878	0.51	151.0559,179.0349,135.0452	$C_{16}H_{18}O_9$	
Sanguiin H4	5.02	634.0819	633.0733	1.99	300.9986,302.0020,633.0715,463.0510	$C_{27}H_{22}O_{18}$	
1-O-p-Coumaroyl-beta-D-glucose	5.21	325.0933	325.0929	1.22	169.0142,145.0294	$C_{15}H_{18}O_8$	
3-O-p-Coumaroylquinic acid	5.96	337.0931	337.0929	0.56	173.0453	$C_{16}H_{18}O_8$	
Gallic acid	6.04	169.0142	169.0143	-0.51	125.0242	$C_7H_6O_5$	
corilagin	6.15	634.0832	633.0733	3.98	465.0669,169.0140,483.0775,313.0564,125.0243	$C_{27}H_{22}O_{18}$	
trans-5-O-(4-coumaroyl)-D-quinic acid	6.63	337.0934	337.0929	1.50	191.0558,192.0600	$C_{16}H_{18}O_8$	
**Amino acids**							
L-Theanine	1.15	174.1004	173.0932	-0.18	173.0931	$C_7H_{14}N_2O_3$	Authentic standard
**Organic acids**							
Ethyl aconitate	4.99	183.0296	201.0405	-1.38	124.0165,183.0293	$C_8H_{10}O_6$	
2,4,6-Trihydroxybenzoic acid	5.77	169.0141	169.0143	-0.58	169.0141,125.0243	$C_7H_6O_5$	
**Proanthocyanidins**							
Prodelphinidin A1	3.79	607.1085	607.1093	-1.44	423.0718,305.0665,125.0243	$C_{30}H_{24}O_{14}$	
3'-Galloylprodelphinidin B2	4.67	761.1341	761.1359	-2.41	423.0718,407.0766,761.1339,609.1234,177.0191	$C_{37}H_{30}O_{18}$	
3,3'-Digalloylprodelphinidin B2	5.38	913.1573		3.38	761.1341,743.1233,125.0243	$C_{44}H_{34}O_{22}$	
(2R,3S,4S)-leucoc	6.78	306.0738	305.0667	-0.39	183.0296,125.0243,161.0243,30	$C_{15}H_{14}O_7$	

yanidin					5.0665	
Epiafzelechin 3-O-gallate-(4beta->6)-epigallocatechin 3-O-gallate	7.18	881.1551	881.1571	-2.17	179.0348,287.0558,269.0455	$C_{44}H_{34}O_{20}$
(-)-Epiafzelechin 3-gallate	8.12	426.0949	425.0878	-0.31	289.0714,183.0296,273.0762,425.0874	$C_{22}H_{18}O_{9}$
**Theaflavins**						
Theaflavin	9.54	563.1189	563.1195	-0.99	125.0244,269.0455,425.0876	$C_{29}H_{24}O_{12}$
**Coumarins**						
Aegelinol	5.74	246.0886	245.0819	-2.35	203.0707,245.0812,187.0397,221.0813,159.0450	$C_{14}H_{14}O_{4}$
Cleomiscosin A	6.12	431.0985	385.0929	0.38	245.0452,417.0832	$C_{20}H_{18}O_{8}$
**Saccharides and glycoside derivatives**						
1-alpha-D-galactosyl-sn-glycerol 3-phosphate	0.77	333.0593	333.0592	0.25	333.0593,241.0112,152.9957,96.9665,78.9587	$C_{9}H_{19}O_{11}P$
3-beta-Gentiobiosylglucose	0.88	504.1683	503.1618	-1.40	191.0557，341.1088,473.1508,503.1609	$C_{18}H_{32}O_{16}$
D-Galactopyranosyl-(1->3)-D-galactopyranosyl-(1->3)-L-arabinose	0.88	519.1566	473.1512	-0.12	191.0557,341.1088	$C_{17}H_{30}O_{15}$
beta-Glucogallin	2.23	331.0670	331.0671	-0.10	169.0142	$C_{13}H_{16}O_{10}$
Glucogallin	2.48	331.0670	331.0671	-0.06	169.0140,125.0242,331.0678	$C_{13}H_{16}O_{10}$
1,2-Digalloyl-beta-D-glucopyranose	2.70	465.0674	483.0780	-0.12	169.0142	$C_{20}H_{20}O_{14}$
1,6-bis-O-galloyl-beta-D-glucose	4.24	483.0774	483.0780	-1.26	169.0141,423.0580,271.0457,177.0194,211.0143	$C_{20}H_{20}O_{14}$
1,6-Digalloyl-beta-D-glucopyranose	4.53	483.0775	483.0780	-1.02	423.0579,169.0143,271.0457,211.0245	$C_{20}H_{20}O_{14}$
1,3,4-Trigalloyl-beta-D-glucopyranose	6.15	636.0977	635.0890	2.31	635.0874,465.0671,169.0141,483.0774,125.0242	$C_{27}H_{24}O_{18}$
**Linalool glycosides**						
Linalool oxide D 3-[apiosyl-(1->6)-glucoside]	7.47	509.2239	463.2185	-0.25	331.1765	$C_{21}H_{36}O_{11}$
Linalool 3,6-oxide primeveroside	8.18	464.2250	463.2185	-1.65	331.1761,463.2178,161.0456	$C_{21}H_{36}O_{11}$
L-Linalool	10.67	448.2304	447.2236	-0.94	315.1816,447.2241	$C_{21}H_{36}O_{10}$

3-[xylosyl-(1->6)-glucoside]						
**Tannins**						
Methyl 6-O-galloyl-beta-D-glucopyranoside	3.65	345.0827	345.0827	-0.19	183.0297,225.0402	$C_{14}H_{18}O_{10}$
Samarangenin A	4.67	759.1184	759.1203	-2.43	423.0718,609.1234,591.1130,289.0715	$C_{37}H_{28}O_{18}$
Samarangenin B	5.83	911.1294	911.1313	-1.99	457.0773,169.0141,125.0243	$C_{44}H_{32}O_{22}$
Methyl 2,3,6-tri-O-galloyl-beta-D-glucopyranoside	6.91	650.1148	649.1046	4.45	169.0142,477.0682,479.0801	$C_{28}H_{26}O_{18}$
**Lipids**						
Gingerglycolipid A	16.34	676.3649	675.3597	-3.09	397.1348,415.1452,277.2171	$C_{33}H_{56}O_{14}$
LysoPC(18:3(9Z,12Z,15Z))	16.59	562.3144	516.3096	-1.15	277.2169,502.2932	$C_{26}H_{48}NO_7P$
2-linoleoyl-sn-glycero-3-phosphocholine	17.09	564.3305	518.3252	-0.35	279.2325,504.3089	$C_{26}H_{50}NO_7P$
1-pentadecanoyl-sn-glycero-3-phosphocholine	17.49	480.3090	480.3096	-1.24	255.2323,480.3091	$C_{23}H_{48}NO_7P$
LysoPC(16:0)	17.49	540.3304	494.3252	-0.60	255.2323,480.3090	$C_{24}H_{50}NO_7P$
LysoPC(18:1(9Z))	17.71	566.3460	520.3409	-0.69	281.2482,506.3243	$C_{26}H_{52}NO_7P$
**Others**						
4-coumaroyltriacetic acid lactone	7.16	272.0684	271.0612	-0.23	125.0243,245.0813	$C_{15}H_{12}O_5$
