# Supplementary material for: Chemical and Quality Analysis of Beauty Tea Processed from Fresh Leaves of Tieguanyin Variety with Different Puncturing Degrees
Source: Foods. 2023 Apr 22;12(9):1737. doi: 10.3390/foods12091737 (PMC10178084; doi:10.3390/foods12091737)
Supplement: Supplementary file 1 [file foods-12-01737-s001.zip › Table S2.pdf]

Table S2. The information on 65 non-volatile differential metabolites in beauty tea.

metabolite name	TR (min)	accurate mass	theoretical mass	ppm	MS/MS ions	formula	Ref.
**Catechins and their derivatives**							
Theacitrin A	6.44	759.1222	759.1203	2.47	169.0143,455.0616,607.1086,483.0573	$C_{37}H_{28}O_{18}$	
(-)-Epigallocatechin	6.79	306.0741	305.0667	0.55	125.0245,124.0167,139.0403,179.0353,305.0665	$C_{15}H_{14}O_7$	
Epicatechin 3-O-(3-O-methylgallate)	8.12	456.1056	455.0984	-0.19	289.0716,183.0297,124.0167	$C_{23}H_{20}O_{10}$	
3,5-Digalloylepicatechin	8.26	593.0934	593.0937	-0.53	441.0833,423.0718,593.0976	$C_{29}H_{22}O_{14}$	
(-)-Epigallocatechin 3,3'-di-gallate	9.06	591.0782	609.0886	0.34	169.0146,287.0199	$C_{29}H_{22}O_{15}$	
Epigallocatechin 3-O-gallate-(4beta->6)-epicatechin 3-O-gallate	6.18	898.1616	897.1520	2.50	745.1390,169.0143,897.1499,727.1284	$C_{44}H_{34}O_{21}$	
Epicatechin 3-O-gallate-(4beta->6)-epigallocatechin 3-O-gallate	7.81	898.1625	897.1520	3.60	169.0144	$C_{44}H_{34}O_{21}$	
Epicatechin-(4beta->8)-epigallocatechin 3-O-gallate	7.82	746.1469	745.1410	-1.94	289.0719,125.0245	$C_{37}H_{30}O_{17}$	
Theasinensin B	9.07	743.1246	767.1829	-0.96	169.0147,591.0901	$C_{37}H_{30}O_{18}$	
Procyanidin A2	9.30	575.1187	575.1195	-1.39	539.1004	$C_{30}H_{24}O_{12}$	
Epigallocatechin-(4beta->8)-epicatechin 3-O-gallate	10.02	727.1301	745.1410	-0.41	593.1288	$C_{37}H_{30}O_{17}$	
**Flavones and flavonols/their glycoside**							
(3''-Apiosyl-6''-malonyl)astragalin	2.27	647.1239	665.1359	-2.23	125.0244,455.0614,437.0512	$C_{29}H_{30}O_{18}$	
Myricetin 3-O-glucoside	6.46	480.0902	479.0831	-0.50	316.0226,318.0315	$C_{21}H_{20}O_{13}$	
2-hydroxy-5-methylquinone	6.79	183.0298	137.0244	-0.47	183.0298,125.0245,168.0065	$C_7H_6O_3$	
Quercetin 3-(2-glucosylrhamnoside)	7.04	609.1452	609.1461	-1.52	609.1446,413.0875,577.1548,301.0348,300.0274	$C_{27}H_{30}O_{16}$	
Plantagoside	7.09	465.1029	465.1039	-2.06	313.0918,151.0040,465.1010	$C_{21}H_{22}O_{12}$	

Isoquercitrin	7.13	463.0846	463.0882	-7.75	300.0273,302.0378	$C_{21}H_{20}O_{12}$	
Oolonghomobisflavan A	7.59	928.1677	927.1626	-2.30	169.0144,573.1033,125.0246	$C_{45}H_{36}O_{22}$	
Isoengelitin	8.27	433.1143	433.1140	0.56	271.0609,125.0246	$C_{21}H_{22}O_{10}$	
Isolariciresinol 4'-O-beta-D-glucoside	8.34	522.2094	521.2028	-1.37	341.1395,279.0877	$C_{26}H_{34}O_{11}$	
Aspalathin	8.59	497.1330	451.1246	6.53	453.1431,497.1328	$C_{21}H_{24}O_{11}$	
Quercetin 3-(6'''-p-coumarylglucosyl)(1->2)-rhamnoside 7-glucoside	8.84	918.2455	917.2357	2.76	289.0719,441.0828,561.1381	$C_{42}H_{46}O_{23}$	
Kaempferol 3-O-caffeoyl-sophoroside 7-O-glucoside	8.87	933.2295	933.2306	-1.25	441.0827,933.2291	$C_{42}H_{46}O_{24}$	
Kaempferol 3-O-[6-(4-coumaroyl)-beta-D-glucosyl-(1->2)-beta-D-glucosyl-(1->2)-beta-D-glucoside]	9.15	458.1149	917.2357	1.56	145.0296,615.1918,285.0404	$C_{42}H_{46}O_{23}$	
Kaempferol 3-neohesperidoside-7-(2''-p-coumaryllaminaribioside)	9.37	531.1441	1063.2936	1.77	755.1819,901.2379,431.0977,145.0295	$C_{48}H_{56}O_{27}$	
2''-(6''-p-Coumaroylglucosyl)quercitrin	9.37	756.1924	755.1829	2.89	285.0403,145.0295,450.1167,301.0351,431.0979	$C_{36}H_{36}O_{18}$	
Kaempferol 2G-coumaroylrutinoside	9.80	739.1861	739.1880	-2.56	285.0404,593.1298,739.1863	$C_{36}H_{36}O_{17}$	
7-O-(4-Hydroxycinnamoyl) astragalin	15.53	593.1293	593.1301	-1.23	593.1303,549.1040,505.1136	$C_{30}H_{26}O_{13}$	
**Phenolic acids**							
Gallic acid	2.25	169.0143	169.0143	0.37	125.0243,124.0167	$C_7H_6O_5$	
Theogallinin	3.48	799.1335	799.1363	-3.51	437.0515,455.0621,153.0192,607.0740	$C_{36}H_{32}O_{21}$	
3-O-p-Coumaroylquinic acid	6.62	337.0934	337.0929	1.45	191.0560	$C_{16}H_{18}O_8$	
Ellagic acid	6.87	300.9991	300.9990	0.41	300.9987,283.9962	$C_{14}H_6O_8$	
**Amino acids**							
L-Theanine	1.25	174.1005	173.0932	0.20	155.0831,173.0931	$C_7H_{14}N_2O_3$	Authentic standa

rd

**Organic acids**						
12-O-Î²-D-Glucopyranosyloxyjasmonic acid	5.95	387.1661	387.1661	0.09	173.0456,387.1657	$C_{18}H_{28}O_9$
Achimilic acid	9.40	555.2233	277.1082	-0.42	507.2021,477.1931	$C_{15}H_{18}O_5$
**Proanthocyanidins**						
3,3'-Digalloylprodelphinidin B2	5.22	456.0706	913.1469	1.77	743.1234,761.1335,137.0245,447.0717	$C_{44}H_{34}O_{22}$
(-)-Epiafzelechin 3-gallate	5.93	851.1814	425.0878	-1.77	289.0717	$C_{22}H_{18}O_9$
Epiafzelechin 3-O-gallate-(4beta->6)-epigallocatechin 3-O-gallate	6.11	927.1576	881.1571	-5.63	591.1128,573.1025,743.1233	$C_{44}H_{34}O_{20}$
Epiafzelechin-(4beta->6)-epicatechin 3,3'-digallate	7.42	866.1722	865.1622	3.22	865.1601,713.1491,695.1391,423.0721,169.0143	$C_{44}H_{34}O_{19}$
**Theaflavins**						
Isotheaflavin 3'-gallate	4.09	761.1338	715.1305	-2.92	591.1130,609.1234,761.1337,453.0823	$C_{36}H_{28}O_{16}$
Theaflavate B	4.55	745.1391	699.1355	-2.71	169.0144,593.1295	$C_{36}H_{28}O_{15}$
Theaflavin 3,3'-digallate	5.40	913.1447	867.1414	-2.57	423.0717,743.1230,761.1342,125.0245	$C_{43}H_{32}O_{20}$
Neotheaflavin 3-gallate	7.23	761.1346	715.1305	-1.86	460.0551,416.0655,727.1296,683.1400,591.1140	$C_{36}H_{28}O_{16}$
Theaflavin	9.53	563.1190	563.1195	-0.88	269.0456,137.0246,425.0872	$C_{29}H_{24}O_{12}$
**Saccharides and glycoside derivatives**						
6-O-acetyl-beta-D-glucose	0.85	267.0719	221.0667	-1.17	191.0559,192.0593,267.0722	$C_8H_{14}O_7$
3-Fucosyllactose	0.86	533.1717	487.1668	-1.27	191.0559,192.0593,473.1505	$C_{18}H_{32}O_{15}$
isomaltulose	0.87	342.1164	341.1089	0.5	191.0558,473.1504,341.1089	$C_{12}H_{22}O_{11}$
D-Gal alpha 1->6D-Gal alpha 1->6D-Glucose	0.87	549.1666	503.1618	-1.20	191.0558,473.1505	$C_{18}H_{32}O_{16}$
1-Hexanol arabinosylglucoside	8.33	377.1818	395.1923	0.30	281.0839	$C_{17}H_{32}O_{10}$
beta-Glucogallin	2.48	331.0672	331.0671	0.26	169.0142	$C_{13}H_{16}O_{10}$
1,2-Digalloyl-beta-D-glucopyranose	4.25	483.0777	483.0780	-0.76	169.0143	$C_{20}H_{20}O_{14}$

1,6-bis-O-galloyl-beta-D-glucose	4.54	483.0777	483.0780	-0.62	169.0144,423.0569,271.0456	$C_{20}H_{20}O_{14}$
khelloside	4.81	407.0981	407.0984	-0.61	245.0452,343.0467,246.0489	$C_{19}H_{20}O_{10}$
Jasmolone glucoside	5.74	387.1661	341.1606	0.06	173.0459,387.1665,163.1132,191.0562	$C_{17}H_{26}O_{7}$
**Terpenoids**						
Linalool oxide D 3-[apiosyl-(1->6)-glucoside]	7.47	509.2240	463.2185	0.08	331.1771	$C_{21}H_{36}O_{11}$
Linalool 3,6-oxide primeveroside	7.69	464.2252	463.2185	-1.3	331.1764,463.2181	$C_{21}H_{36}O_{11}$
Linalool 3,7-oxide beta-primeveroside	8.17	464.2252	463.2185	-1.26	331.1761,463.2178,161.0456	$C_{21}H_{36}O_{11}$
L-Linalool 3-[xylosyl-(1->6)-glucoside]	10.66	448.2304	447.2236	-0.94	315.1821,447.2246	$C_{21}H_{36}O_{10}$
**Tannins**						
Theaflavic acid	3.71	473.0726	427.0671	0.05	167.0355,319.0459,439.0671	$C_{21}H_{16}O_{10}$
Methyl 2,3,6-tri-O-galloyl-beta-D-glucopyranoside	6.91	650.1145	649.1046	4.03	169.0142,125.0246,649.1032	$C_{28}H_{26}O_{18}$
Samarangenin A	7.81	759.1194	759.1203	-1.15	727.1288,169.0143,575.1183,289.0719,589.0984	$C_{37}H_{28}O_{18}$
**Lipids**						
Diosbulbinoside F	6.61	538.2049	537.1978	-0.25	191.0561,491.1915	$C_{26}H_{34}O_{12}$
LysoPC(18:1(9Z))	17.71	566.3446	520.3409	-3.31	281.2484,506.3251	$C_{26}H_{52}NO_{7}P$
(1beta,2alpha,3alpha)-1,2,3,24-Tetrahydroxy-12-oleanen-28-oic acid	18.03	485.3274	503.3378	0.21	353.2126,485.3273,163.1132	$C_{30}H_{48}O_{6}$
**Others**						
Phaeophorbide b	18.31	605.2398	605.2406	-1.26	529.2244	$C_{35}H_{34}N_{4}O_{6}$
