# Supplementary material for: Chemical and Quality Analysis of Beauty Tea Processed from Fresh Leaves of Tieguanyin Variety with Different Puncturing Degrees
Source: Foods. 2023 Apr 22;12(9):1737. doi: 10.3390/foods12091737 (PMC10178084; doi:10.3390/foods12091737)
Supplement: Supplementary file 1 [file foods-12-01737-s001.zip › Table S3.pdf]

Table S3. The volatile components with OA Vs ≥ 1.

No.	Compounds	OT (μg/kg)	Odour Description	OAVs		
				NPBT	LPBT	HPBT
1	1,6-octadien-3-ol, 3,7-dimethyl-	0.006	floral, fruity	3758.33	3876.67	3828.33
2	1,5,7-octatrien-3-ol, 3,7-dimethyl-	0.65	floral, fruity	66.32	94.69	160.09
3	geraniol	0.0066	floral, sweet	2240.91	1710.61	1410.61
4	cedrol	0.5	cedarwood-like	2.18	-	-
5	cis-linalool oxide	0.32	sweet, floral,cream	38.34	48.75	95.25
6	benzeneacetaldehyde	0.0063	floral, fruity, sweet	798.41	473.02	1003.17
7	decanal	0.1	sweet, floral, citrus-like	44.7	52.3	54.6
8	1-cyclohexene-1-carboxaldehyde, 2,6,6-trimethyl-(β-cyclocitral)	0.003	fruity, fresh and sweet	813.33	1466.67	1443.33
9	methyl salicylate	0.04	holly oil, minty	163.5	149.25	152
10	furan, 2-pentyl-	4.8	fruity, green	-	2.45	-
